# ROS‐responsive drug delivery systems

**DOI:** 10.1002/btm2.10014

**Published:** 2016-07-05

**Authors:** Jing Liang, Bin Liu

**Affiliations:** ^1^ Dept of Chemical and Biomolecular Engineering National University of Singapore 117585 Singapore; ^2^ Institute of Materials Research and Engineering, Agency for Science, Technology and Research (A*STAR) Innovis Singapore 138634

**Keywords:** chemotherapy, drug delivery system, reactive oxygen species, ROS‐responsive, stimuli‐responsive, theranostics

## Abstract

Reactive oxygen species (ROS) play an important role in signal transduction and metabolism. Over‐produced ROS in cells or tissues, however, often leads to oxidation stress that has implications in a series of diseases including cancer, aging, atherosclerosis and inflammation. Driven by the need for on‐demand drug delivery and fuelled by recent development of ROS‐responsive materials and nanomedicine, responsive drug delivery systems (DDSs) have gained increasing research interest. ROS‐responsive DDS is designed to release therapeutic agents only in targets of interest that produce excessive ROS, which may lead to both enhanced therapeutic efficiency and reduced side effects. Multiple‐stimuli responsive DDSs that are also sensitive to other stimuli can further enhance controlled drug release in sites where multiple stimuli coexist. Beyond drug delivery, multifunctional DDSs have great potential in achieving simultaneous imaging, combinatorial therapy and targeting ability by introducing multifunctional elements such as signal reporter, targeting elements and photosensitizer. This review will summarize the latest development of ROS‐responsive DDSs and discuss their design principle and biomedical applications.

## 1 INTRODUCTION

Tremendous efforts have been devoted for the development of drug delivery systems (DDSs) that can effectively deliver therapeutic agents into disease sites. However, therapeutic efficiency is often hampered by premature drug release and rapid body clearance, which not only requires large dose of drug but also causes unwanted systemic toxicity.^1^ On‐demand drug delivery is thus of utmost importance in achieving site‐specific delivery with reduced side effects. In view of this, stimuli‐responsive DDSs have gained increasing popularity due to their ability to release payload only in response to a specific stimulus that is associated with certain disease conditions. Typical stimuli explored by DDSs include endogenous (e.g., reactive oxygen species (ROS), redox, pH, thermal and enzyme) and exogenous (e.g., light, temperature, magnetic field and ultra sound) ones.^2^ Of particular interest is the ROS‐responsive DDSs because ROS overproduction has great implications on a variety of diseases and emerging ROS‐responsive materials hold great promise in the development of advanced nanomedicines. An effective DDS relies on both a solid understanding of physiological conditions of the disease sites and rational design of stimuli‐responsive drug carrier that can undergo sharp chemical or physical changes in response to the stimuli to allow for cargo escape. Certain pathological conditions with coexisting multiple stimuli are better targeted by multiple‐stimuli responsive DDSs to enable improved drug efficacy. With the help of a photosensitizer that can produce ROS on light irradiation, controlled drug delivery can be theoretically achieved in any site of interest, which further expands the application for treatment of a wide range of diseases.

ROS are generally referred to a class of oxygen derived chemical species produced by the body. Typical ROS species include hydrogen peroxide (H_2_O_2_), singlet oxygen (^1^O_2_), superoxide ( $O_{2^{•}}^{-}$) and hydroxyl radicals (HO•), which may transform from one to another via a cascade of reactions.^3^ They can be generated endogenously from mitochondrial metabolism or NADPH enzyme catalyzed reactions as well as exogenously by exposure to UV light or xenobiotic compounds.^4^ While moderate level of ROS plays a vital role in physiological and pathological processes,^5^ overproduction of ROS that overwhelms the antioxidant defense system will lead to oxidative stress. ROS usually contain unpaired electrons or unstable bonds, which make them highly reactive. Prolonged exposure to high level ROS will cause irreversible functional alterations or complete damage to nucleic acid, proteins, lipid, and hydrocarbons. The toxic effect of excessive ROS is thus associated with an array of pathological conditions, including cancer,^6^ aging,^7^ diabetes,^8^ cardiovascular diseases,^9^ and neurodegenerative diseases.^10^

**Scheme 1:**
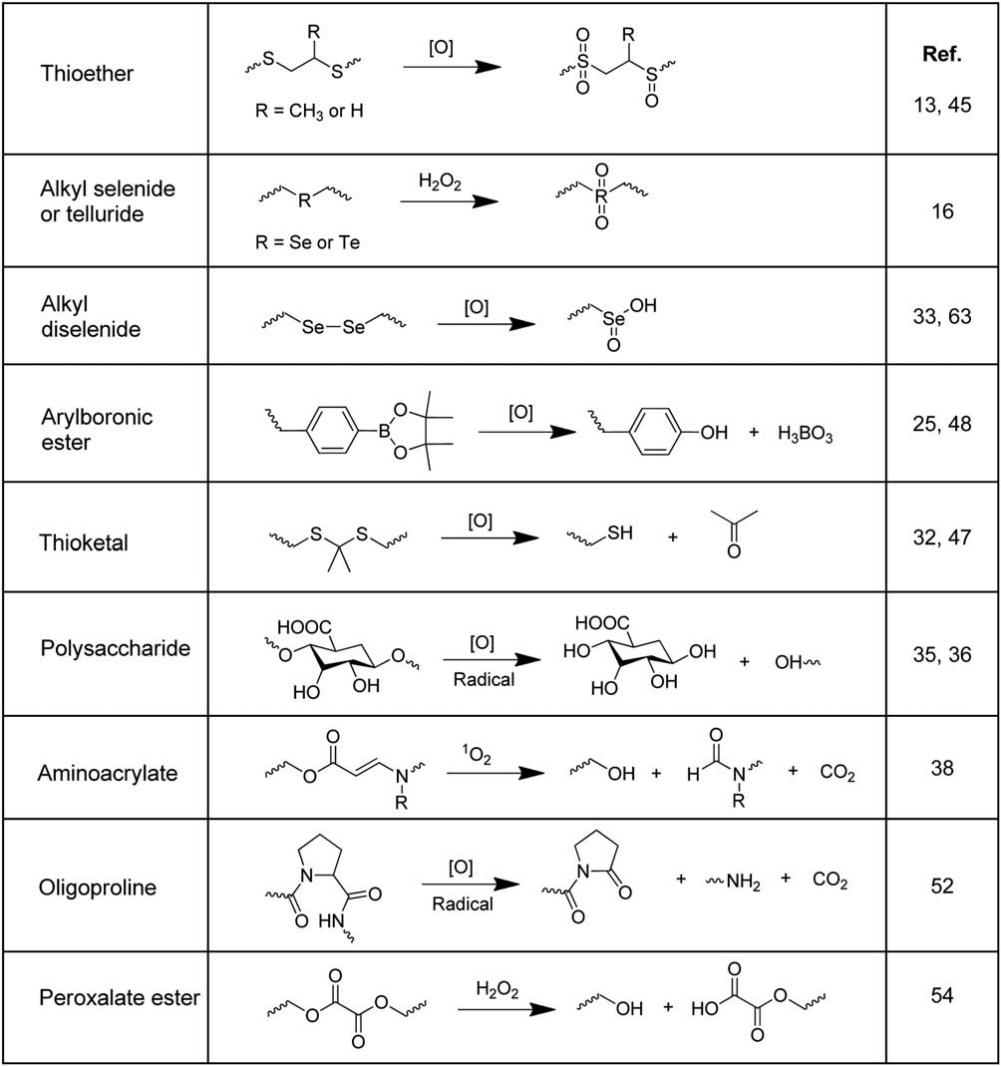
Reaction scheme of ROS‐responsive materials for drug release

There are many types of ROS‐responsive materials explored in drug delivery applications, including those containing thioether, selenium/tellurium, thioketal, polysaccharide, aminoacrylate, boronic ester, peroxalate ester and polyproline. The reaction mechanism of each type of material is summarized in Scheme 1, which will be discussed in respective sections. Depending on the material or design of the carrier, the major mechanism of drug release can be attributed to solubility change induced carrier disassembly, cleavage induced carrier degradation and carrier‐drug linker cleavage. The detailed synthesis and oxidation properties of most ROS‐responsive materials have been covered in previous review.^11^ In this review, we will discuss the progress of ROS‐responsive DDS, with a focus on the design principles for various applications of DDSs with different stimuli responsiveness.

## 2 SINGLE‐STIMULI RESPONSIVE DDSs

### 2.1 Thioether‐containing polymers

Thioether‐containing polymers are known to exhibit phase transition from hydrophobic to hydrophilic states under oxidative environments. Specifically, the hydrophobic poly(alkylene sulfide)s can be oxidized into more hydrophilic poly(alkylene sulfoxide) and ultimately poly(alkylene sulfone) (Scheme 1).^12^ The early report of oxidation‐responsive polymeric vesicles containing poly(eththelyene sulfide) in 2004 by Hubbell paved the way for development of ROS‐responsive nanocarriers for DDS and biosensing applications.^12^ Drug carriers containing thioethers can be easily degraded on the phase transition, leading to release of their payloads.

Inspired by Hubbell's work, Duvall's group developed ROS‐responsive poly(PS‐b‐DMA) micelles for triggered drug release in 2012.^13^ The micelle drug carrier consists of an amphiphilic diblock copolymer (poly(PS_74_‐b‐DMA_310_)) of propylene sulfide (PS) and *N,N*‐dimethylacrylamide (DMA), which are then loaded with hydrophobic fluorescent indicators Nile red and DiO (3,3′‐dioctadecyloxacarbocyanine perchlorate) as model drugs. The drug loaded carrier was found to be responsive to oxidants including H_2_O_2_ and oxidation leads to solubility change and subsequent dissociation of the nanocarriers to release the model drugs. It took ∼40 hr to release 80% of Nile red at H_2_O_2_ concentration of 1.66%. In addition, DiI (1,1′‐dioctadecyl‐3,3,3′,3′‐tetramethylindocarbocyanine perchlorate) and DiO, a Förster Resonance Energy Transfer (FRET) fluorophore pair was coloaded in the nanocarrier and demonstrated efficient drug release triggered by endogenous oxidants generated in lipopolysaccharide (LPS) treated RAW 264.7 macrophages. Recently, the group explored a poly (propylene sulfide) (PPS) microsphere‐based DDS for in vivo ROS‐demanded drug delivery.^14^ Curcumin, an anti‐inflammatory and antioxidant drug, was encapsulated in PPS microsphere through oil‐in‐water emulsion. The curcumin‐PPS microspheres were demonstrated for in vivo drug delivery for treatment of diabetic peripheral arterial disease, an inflammatory condition arising from elevated level of ROS. Due to combined ROS scavenging effect of PPS and therapeutic effect of curcumin, the DDS enabled accelerated recovery of hind limb ischemia of the diabetic mouse.

### 2.2 Selenium‐ or tellurium‐containing polymers

Similar to sulfur‐containing polymers, compounds containing selenium (Se) and tellurium (Te) which are also from the chalcogen group, were exploited for drug delivery applications as well. The organoselenium and organotellurium compounds can be oxidized from divalent to tetravalent states, making them attractive ROS scavengers.^15^ ROS oxidation of monoselenium and monotellurium compounds may lead to phase transition from hydrophobic to hydrophilic (Scheme 1), which can be capitalized in construction of ROS‐responsive drug carriers.

Early in 2010, Xu and Zhang synthesized an amphiphilic block copolymer (PEG‐PUSe‐PEG) with a hydrophobic monoselenide‐containing block polymer and two hydrophilic blocks of PEG.^16^ The polymer self‐assembles into micelles with an average diameter of 71 nm. On oxidation, the polymer micelles undergo a hydrophobic‐to‐hydrophilic phase transition which causes disassembly of the micelles. It was found that the PEG‐PUSe‐PEG polymer is more sensitive to oxidation stimuli than its sulfur analogue PEG‐PUS‐PEG: almost complete conversion was achieved for the former on exposure to 0.1% H_2_O_2_ for 5 hr while only ∼30% was converted for the latter. These polymer micelles were successfully demonstrated for drug release using doxorubicin (DOX) as the model drug. The drug release profile reaches equilibrium after 10 hr with final DOX release percentage of ∼72%. Later, the group reported a Se‐containing poly(ethylene oxide‐b‐acrylic acid) block copolymers with reversible self‐assembly and disassembly properties on subject to repeated cycles of ROS or Vitamin C exposure.^17^ Another work was also reported by the group based on Se‐containing polymeric superamphiphile.^18^ In 2013, Huang and Yan reported a ROS‐responsive nanocarrier using a Se‐containing amphiphilic hyperbranched polymer micelle.^19^ The polymer consists of hydrophobic selenide side chains and hydrophilic dendritic backbone with phosphate segments. The micelles loaded with DOX were demonstrated for drug release in HeLa cells.

Tellurium‐containing compounds are thought to have higher sensitivity due to the lower electronegativity and lower toxicity than their selenium counterparts,^20^, ^21^ making them attractive drug carriers. The higher oxidation sensitivity of telluride was verified by comparing the oxidation behaviors of telluride, selenide and sulfide dicarboxylic acids using cyclic voltammetry, indicating telluride is a better candidate as drug carrier.^22^ Inspired by selenide containing materials, Xu's group further developed a number of ROS‐responsive systems based on Te‐containing polymers. For example, coassembly of a hydrophobic Te‐containing polymer and phospholipids were demonstrated to have reversible redox responsiveness.^23^ The amphiphilic phospholipid not only aids coassembly formation, but also provides good biocompatibility and degradability. Owing to the reversible redox nature of the Te‐containing polymer, the coassemblies can be oxidized by dilute H_2_O_2_ solution and reduced by ascorbic acid. The polymer can be oxidized in 1 hr in the presence of 100 µM H_2_O_2_. The ROS oxidation, however, did not result in significant morphological changes. A Te‐containing polymer micelle system was also reported to be responsive to both H_2_O_2_ and 2 Gy gamma radiation which leads to NP swelling and dissociation.^22^ Another example demonstrated that the Te‐containing herperbranched polymer aggregates can swell under biologically relevant concentration of H_2_O_2_ due to solubility switch of Te components.^21^

### 2.3 Boronic ester‐containing polymers

Boronic ester, particularly arylboronic acid pinacol ester, has been frequently employed in drug delivery applications due to their ability to be oxidized by H_2_O_2_ at physiological pH and temperature to produce phenol and pinacol borate (Scheme 1).^24^ Two examples of polysaccharide modified at their hydroxyls with arylboronic ester groups have been demonstrated for drug delivery based on a solubility switch strategy. Once modified with boronic ester, the water soluble polysaccharide becomes organic soluble, which facilitates payload encapsulation. Upon exposure to ROS species and oxidation of boronic esters, the polysaccharides are converted back to the water soluble parent form, which concurrently release the payloads. In one example, an oxidation sensitive dextran‐boronic ester conjugate was synthesized for vaccination application.^25^ The modified dextran was designed to encapsulate ovalbumin (OVA), a widely used antigen for immunization. Upon uptake by phagosomes of antigen‐presenting cells (APCs), the OVA loaded nanoparticles (NPs) are degraded by the ROS heavily produced in APC, leading to OVA release. Results showed that OVA loaded NPs increased antigen presentation to CD8^+^ T‐cells by 27‐fold as compared to the non‐responsive NPs.

**Figure 1:**
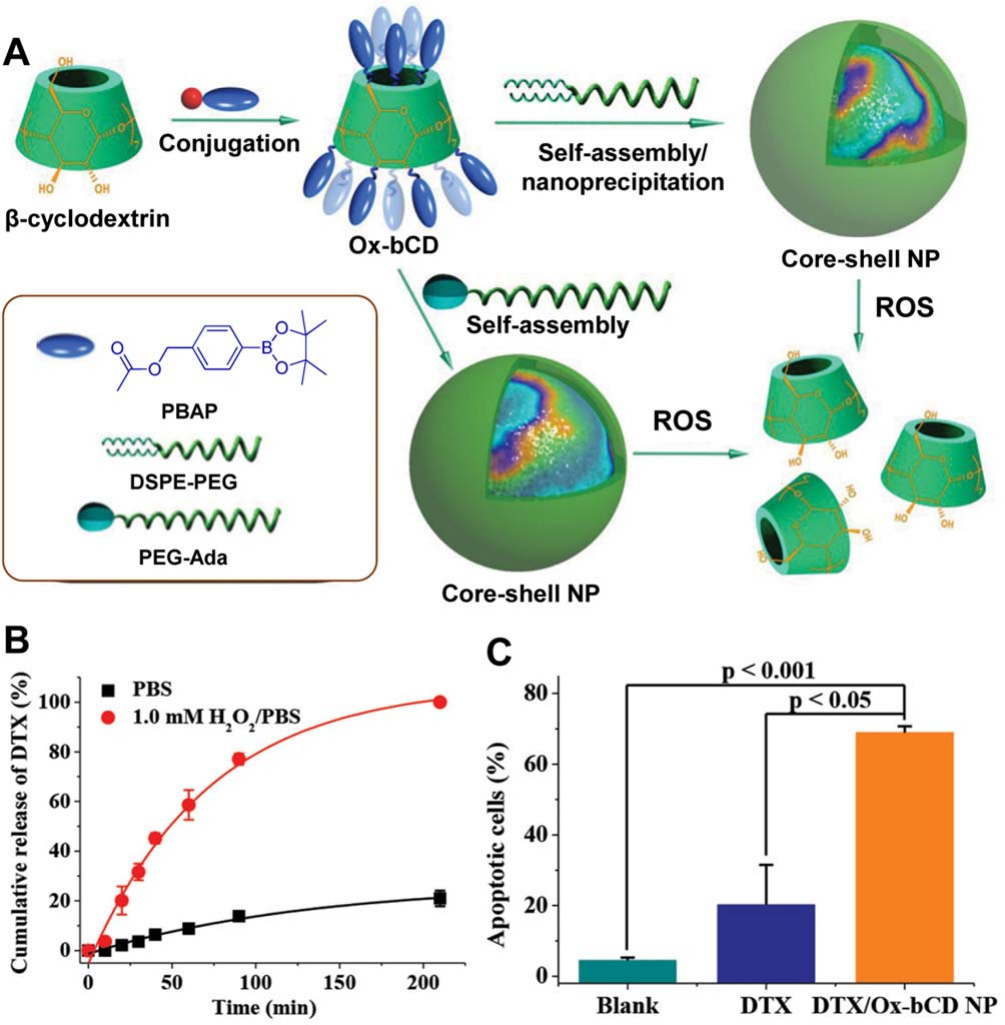
(A) Schematic illustration of fabrication of ROS‐responsive NPs and subsequent oxidation by ROS to parent cyclodextrin. (B) In vitro release profiles of DTX/Ox‐bCD NPs after incubation with mouse melanoma B16F10 cells. (C) Statistical analysis of apoptotic cell percentage of B16F10 cells treated with different samples at [DTX] = 10 μg mL^−1^. Adapted from Ref. 26 with permission. Copyright 2015 American Chemical Society

In another example, β‐cyclodextrin conjugated with boronic ester (Ox‐bCD) was demonstrated for drug delivery in both in vitro and in vivo model.^26^ As shown in Figure 1A, core shell NPs were formed between Ox‐bCD and poly(ethylene glycol)‐ distearoylphosphatidylethanolamine (DSPE‐PEG) or PEG‐adamantyl (Ada) via assembly/nanoprecipitation method taking advantage of hydrophobic interaction between the boronic segments of Ox‐bCD and DSPE and guest‐host interaction between β‐cyclodextrin and Ada, respectively. The NPs are highly biocompatible and are further loaded with a hydrophobic antimitotic chemotherapy drug docetaxel (DTX) to examine in vitro / in vivo drug release. Figure 1B shows that DTX can be completely released within 4 hr upon exposure to 1.0 mM H_2_O_2_, while only 21% of drug was released in the absence of H_2_O_2_. The percentage of apoptotic cells upon incubation with saline (blank control), free DTX and DTX/Ox‐bCD NPs were found to be 4.5%, 20.2%, and 69.0%, respectively (Figure 1C). The tumor volume and body weight of xenograft‐bearing nude mice were studied for 12 days after intravenous administration of DTX loaded NPs and various control samples. The DTX loaded NPs exhibited much higher antitumor efficiency with little effect to body weight, indicating their therapeutic advantage and safety as in vivo DDS.

In comparison to solubility switch approach, aryl boronic ester can also be incorporated into polymers which backbone can be degraded in response to ROS. In one example, boronic esters are introduced to each motif of a polymer that forms NP.^27^ In the presence of H_2_O_2_, boronic ester is cleaved, followed by quinone methide rearrangement to degrade the polymer into smaller pieces. Such a design has an enhanced ROS sensitivity as each boronic ester hydrolysis leads to degradation of polymer backbone. The system has been demonstrated for drug release using Nile red as indicator. In another work, the authors have developed a general strategy to fabricate on‐demand drug delivery based on a chain shattering approach.^28^ The polymer is constructed with alternating trigger‐responsive domains (TRDs) and drugs both on the backbone. The protecting group in the TRD can either be a UV‐responsive *O*‐nitrobenzyloxy‐l‐carbonyl group or a ROS‐responsive boronic ester group. Upon exposure to stimuli, the protecting group is removed to induce elimination reactions along the backbone, leading to shattered polymer and release of drug molecule. NPs were prepared using the ROS‐responsive polymer and poly(ethylene glycol)‐block‐poly(l‐lactide) (PEG113‐b‐PLLA) by nanoprecipitation method. Upon subcutaneous injection of the NPs, the mouse tumor treated with H_2_O_2_ showed a 2.5‐fold higher apoptosis index as compared the one without H_2_O_2_ treatment. Recent work also reported a charge reversal approach for gene delivery based on a boronic acid containing polymer.^29^

### 2.4 DDS with thioketal linkers

Thioketal linkers have been frequently used in recent years as they can be readily cleaved by ROS oxidants, producing ketones and thiols (Scheme 1).^30^ Thioketal linkers are employed in a number of examples to deliver therapeutic agents to inflammation sites or cancer cells that are rich in ROS species. For example, a direct complexation between a cationic poly(amino thioketal) (PATK) and negatively charged DNA was used to deliver gene to prostate cancer cells.^31^ In vitro experiment shows H_2_O_2_ concentration dependent PATK degradation rate and ∼60 hr is needed for 80% degradation in the presence of 100 mM H_2_O_2_. The cleavage of thioketal linkers by elevated levels of ROS in cancer cells results in efficient intracellular release of DNA, leading to significantly higher gene transfection efficiency as compared to the non‐degradable counterparts. To achieve cell targeted gene delivery, the NP complex was further conjugated with a GRP78‐binding peptide, which leads to a three‐fold higher cell uptake efficiency by PC3 cells and two‐fold higher gene transfection efficiency. In another example, a thioketal‐containing polymer formed NP with RNA and cationic lipid complex were used for targeting inflamed intestinal tissues through oral delivery.^32^ This polymer is stable against acid‐, base‐ and enzyme‐catalyzed reactions and can survive the harsh environment of gastrointestinal tract to deliver locally to intestinal tissue that overproduces ROS. These are typical examples of cleavage induced carrier degradation to release the payload encapsulated via electrostatic interaction. Other examples which use thioketal as a linker between the carrier and drug will be covered in later sections.

## 3 MULTIPLE‐STIMULI RESPONSIVE DDSs

### 3.1 ROS‐ and light‐responsive DDSs

Light is one of the most commonly used external stimuli to trigger drug release or therapy activation. In contrast to internal stimuli which may introduce additional complexity and instability, light triggered drug release provides a more reliable spatiotemporal control of release with ease of operation. When integrated with ROS‐responsive DDS, a photosensitizer (PS) is usually used as a light‐sensitive element to generate ROS, mainly singlet oxygen (SO), which in turn activates the ROS‐triggered drug delivery. As the SO generated in excess has cytotoxicity to cells or tissues, the integrated system can be potentially applied for photodynamic therapy (PDT) to result in enhanced therapeutic efficiency. Use of light source in higher wavelengths, especially in the near infrared (NIR) range, is beneficial in achieving non‐invasive therapy with improved tissue penetration.

**Figure 2:**
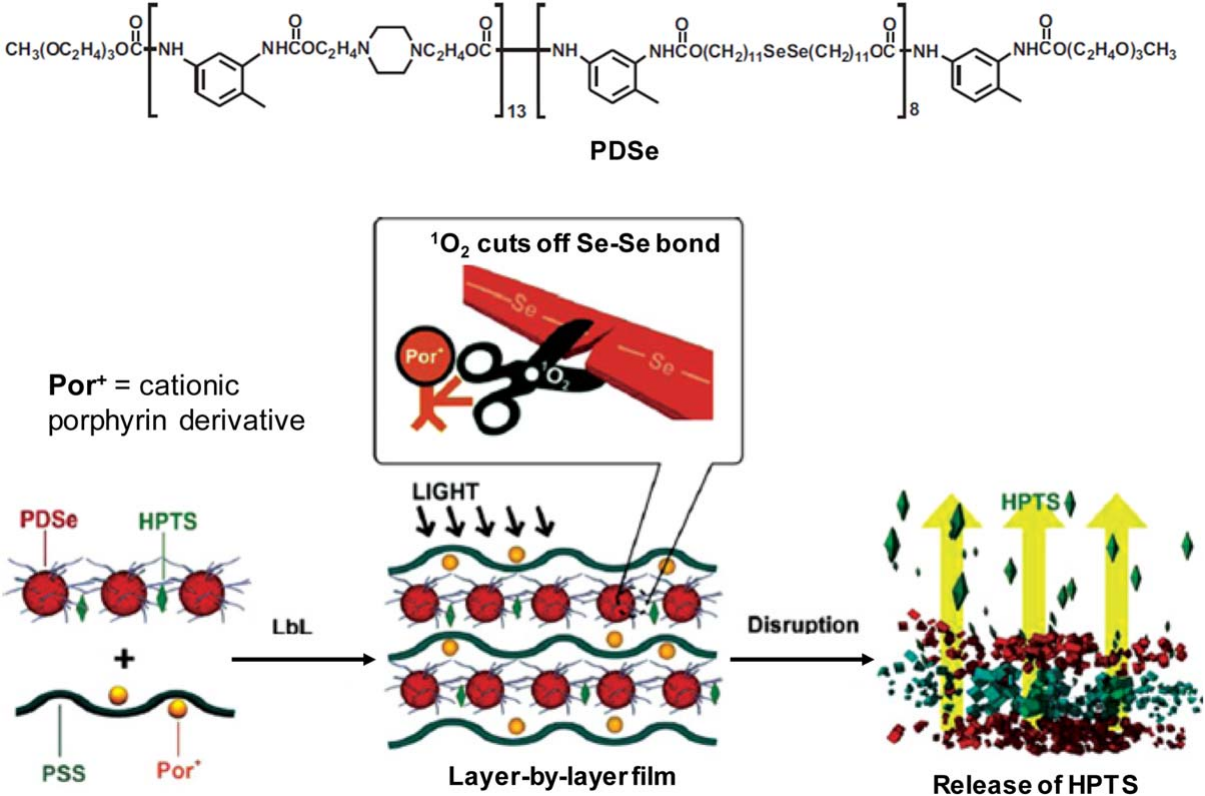
Molecular structure of PDSe and schematic illustration of layer‐by‐layer assembly formation and photochemical cleavage of Se—Se bond induced cargo release. Adapted from Ref. 34. Copyright 2013 Wiley‐VCH Verlag GmbH & Co. KGaA, Weinheim

Different from monoselenides which undergo solubility switch upon ROS oxidation, diselenides can be rapidly cleaved by ROS (Scheme 1), which allows disruption of diselenide‐containing drug carriers. ROS‐ and light‐responsive DDSs have been explored using diselenides polymers with the help of PS as a source of ROS. In one example, triblock copolymer micelles (PEG‐PUSeSe‐PEG) with different PEG lengths were tested for ROS‐responsiveness with addition of a typical PS, a porphyrin derivative 9,10‐anthracenedipropionic acid (ADPA).^33^ It was found that SO released by ADPA upon red light illumination can effectively cleave Se‐Se bond, releasing the DOX encapsulated by the polymer micelles. The drug release efficiency was higher for micelles with shorter PEG chains due to better penetration of SO to react with Se‐Se bond. In another example, the group demonstrated visible light responsive diselenide‐containing layer‐by‐layer films for potential application of combinational chemotherapy and PDT.^34^ As shown in Figure 2, the layer‐by‐layer assemblies consist of a cationic backbone chain containing diselenide (PDSe), alternatively deposited with an anionic complex of oppositely charged poly(styrene sulfonate) (PSS) and porphyrin derivative. A fluorescent indicator cargo 8‐hydroxypyrene‐1,3,6‐trisulfonic acid trisodium salt (HPTS) was also loaded into the polymer film to monitor cargo release. Under visible light irradiation, ROS generated in situ by phorphyrin can cleave the Se‐Se bonds, leading to polymer films disruption and controlled release of cargo. By measuring the fluorescence increase of the HPTS released into the immersing media, the release percentage was found to be as high as ∼80% after 5 hr visible light irradiation. Importantly, part of the ROS generated may also be used for killing of cells or bacteria, making such a system useful for dual modal therapy applications.

Polysaccharides are reported to be depolymerized by ROS species (Scheme 1), which make them promising ROS‐activatable carriers for therapeutic agents. For example, hyaluronic acid (HA)‐Chlorin e6 (Ce6) conjugate has been used for ROS‐triggered PDT and fluorescence imaging.^35^ The fluorescence of the PS Ce6 is quenched when forming NPs. Excess ROS can depolymerize HA to recover fluorescence and concurrently cause phototoxicity to cells. PS is believed to undergo two types of reactions—via electron transfer to generate type I (e.g., O^−^_2_•, HO•) ROS and/or via energy transfer to generate type II ROS (e.g., SO)—upon light irradiation. While most ROS‐responsive DDSs covered are sensitive to type II ROS, type I ROS species are prevalently generated under hypoxic conditions. The electron rich chondroitin sulfate (CS) is known to promote generation of type I ROS, making it a suitable ROS‐degradable carrier under hypoxic conditions. By decorating a PS on CS backbone, a DDS responsive to both external (light) and internal (tumor hypoxia) stimuli was constructed.^36^ The DDS consists of a CS‐PS (pheophorbide) conjugate which forms NPs with loaded DOX. Under laser light illumination, type I ROS was generated along CS backbone at low oxygen level, which depolymerized the CS to cause disassembly of NPs to release DOX. The drug release efficiency increased from 33% to 84% upon light irradiation. The drug loaded NPs showed higher toxicity in colon cancer cells (HCT‐116) under hypoxic condition than that under normoxic condition and exhibited high anti‐tumor efficiency in vivo.

Early in 2008, the cleavage of olefin linkage by SO through 2 + 2 cycloaddition reactions has been employed in construction of light‐responsive prodrug.^37^ Due to the existence of competition reaction and limited reaction yield, You's group has proposed a new linker aminoacrylate (AA) (Scheme 1) to construct ROS‐ and light dual‐responsive materials based on “click and photo‐unclick chemistry.”^38^ Among a number of linkers tested, AA was more effectively cleaved by SO generated upon excitation of long wavelength light (690 nm). Based on this novel system, the same group has developed a series of light‐activatable DDS. One work proposed double activation of prodrugs containing both an AA linker and a PS deactivated by cellular esterase.^39^ Upon cell uptake, the PS is first activated by esterase and which then releases drugs via photounclick chemistry by visible light (540 nm). Coumarin prodrug was successfully demonstrated for effective drug delivery (99% efficiency) after incubation with MCF‐7 cells for 30 min upon light irradiation. The group also demonstrated the first example of low energy activated prodrug using the photounclick chemistry.^40^ Due to the ability of the prodrug to be activated with far‐red light (690 nm), the prodrug system was applied in mouse model, showing enhanced anti‐tumor efficacy than that using its noncleavable analogue.

Based on a similar design principle, the group further developed prodrug systems for combined controlled drug delivery and PDT as well as bioimaging. The prodrug Pc‐(L‐CA4)_2_ is comprised of a cytotoxic drug combretastatin A‐4 (CA4), a ROS cleavable AA linker (L) and two PS moieties of phthalocyanine (Pc). The prodrug showed lower toxicity than the free drug but exhibited improved cytotoxicity under far‐red light illumination. Fluorescence change was monitored to reveal the drug distribution over time. Antitumor efficacy was tested for tumor bearing mouse injected with the prodrug subject to light illumination. It was found that tumor volume shrank to nonmeasureble size after 24 hr light irradiation and remained so throughout 15 days, indicating effective tumor ablation, which is presumably due to the combined effect of PDT and local chemotherapy effect.^41^ Based on this work, the group further introduced a tumor‐targeting group folic acid (FA) and PEG segment in the prodrug CA_4_‐L‐Pc‐PEG_n_‐FA and investigated the PEG length on the antitumor efficacy.^42^ Results show that the prodrug with longer PEG chain demonstrated more specific uptake by tumors and resulted in higher tumor ablation efficiency than those with shorter PEG chains as well as the prodrug without FA.

**Figure 3:**
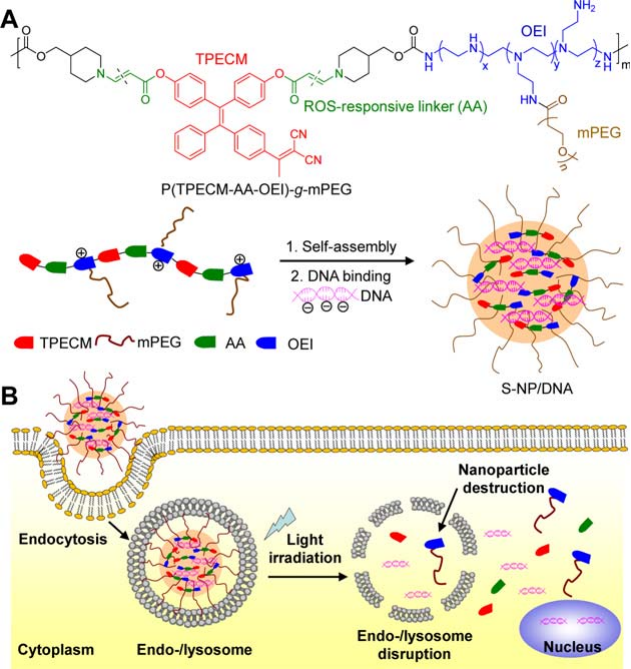
(A) Chemical structure of the ROS‐responsive polymer P(TPECM‐AA‐OEI)‐*g*‐mPEG and complexation of the polymer and DNA to form ROS‐responsive NPs. (B) Schematic illustration of endocytosis of ROS‐responsive NPs and light triggered endo/lysosome escape and DNA unpacking. Adapted from Ref. 44 with permission. Copyright 2015 Wiley‐VCH Verlag GmbH & Co. KGaA, Weinheim

In addition to the commonly used porphyrin based PS, other fluorescent materials with photosensitivity were also explored in ROS‐responsive DDS. Fluorogens with aggregation‐induced emission (AIE) are a unique class of materials that are only strongly emissive in solid or aggregated state.^43^ PS based on AIE aggregates show high signal‐to‐noise ratios and efficient ROS generation. Recently, Liu's group has proposed a novel photoactivatable system for light‐controlled gene delivery using an AIE‐active polymer as PS.^44^ Endo/lysosome escape of gene vectors and subsequent unpacking in cytosol represent two major challenges in efficient gene delivery. ROS‐responsive systems are especially useful in achieving controllable gene unpacking and release. As shown in Figure 3A, the polymer consists of an AIE PS (TPECM) conjugated with DNA‐binding low molecular weight oligoethylenimine (OEI) via an AA linker with hydrophilic PEG side chains. OEI was used as it is believed to facilitate endo/lysosomal escapes via “proton sponge effect” and it has lower toxicity as compared to its high molecular weight counterpart. The polymer then forms complex with negatively charged DNA to form highly emissive water soluble NPs. Upon cell uptake of the NPs through endocytosis and subsequent light irradiation, ROS is generated by PS to disrupt endo/lysosomal membranes to allow for vector escape. Concurrently, the generated ROS can break the AA linker to degrade polymer into smaller components, leading to unpacking of nucleic acids. The NPs were tested in different types of cell lines including MCF‐7, HeLa, HepG2, A549, HEK293, and NIH 3T3 cells for transfection efficiency and the average showed over 50% increase as compared to the commercial PEI_25K_. The strategy proposed can be generalized to deliver other types of therapeutic drugs. A similar work was reported recently using a Ce6 conjugated PPS for DOX deliver and endo/lysosomal escape.^45^ Taking advantage of the light‐up characteristics of AIE probe, another work was reported for tracking the activation of PDT *via* ROS‐triggered fluorescence turn on.^46^

DDS that combines diagnostic, therapeutic and targeting functions in a single platform are highly desirable for biomedicine. In this regard, Liu's group has developed an integrated system based on thioketal‐containing conjugated polyelectrolytes (CPEs) for combined PDT and controlled drug delivery with targeting ability.^47^ CPEs are known for their high absorption and emission efficiencies and some of them exhibit photoactivity which can generate ROS under light illumination. In this study, a photosensitive CPE of PFVBT serves as a fluorescent indicator, a PS and a drug carrier. The side chains of the CPEs contain PEG segments with covalently linked DOX via ROS cleavable thioketal linker. The obtained prodrug self‐assembles into NPs, which are then functionalized with cyclic arginine‐glycine‐aspartic acid tripeptide (cRGD) to yield RGD‐CP‐DOX NPs for targeting α_v_β_3_ integrin overexpressed cells. Upon cancer cell internalization and subsequent light irradiation, the NPs can generate ROS for PDT and ROS will cleave the thioketal linker to release drugs. By monitoring the fluorescence of the polymer, it was shown that the NPs can target preferentially to MDA‐MB‐231cells that have high α_v_β_3_ integrin expression level over MCF‐7 cells that have low α_v_β_3_ integrin expression levels. In addition, RGD‐CP‐DOX NPs were found to cause significantly higher cell apoptosis or cell death with light exposure as compared to that without light exposure or the control RGD‐CP NPs. The results indicate the combined effect of PDT and ROS‐triggered drug delivery that contribute to the enhanced therapeutic efficiency.

### 3.2 ROS and Enzyme dual/multiple stimuli responsive DDSs

Dual responsiveness to both ROS and enzymes are beneficial for DDS in achieving enhanced therapeutic effects as both stimuli are commonly coexistent in pathological conditions such as tumor and inflammation. In such a dual‐responsive system, an enzyme cleavable substrate is usually incorporated to modulate drug release. For example, an amphiphilic block copolymer was designed to enable drug release in response to both ROS and matrixmetalloproteinase‐2 (MMP‐2).^48^ The hydrophobic block of polymer consists of an inactive MMP‐2 attached to the backbone via a boronic ester linkage while the hydrophilic block comprised of an MMP‐2 peptide substrate (GPLGLAGGERDG). In inflammatory environment, the over‐secreted MMP‐2 cleaves the peptide substrate to cause a morphology change of the polymer from miceller NPs to micron‐scale aggregates. In the meantime, ROS also cleaves the boronic ester to release MMP‐2 inhibitor for anti‐inflammation therapy.

**Figure 4:**
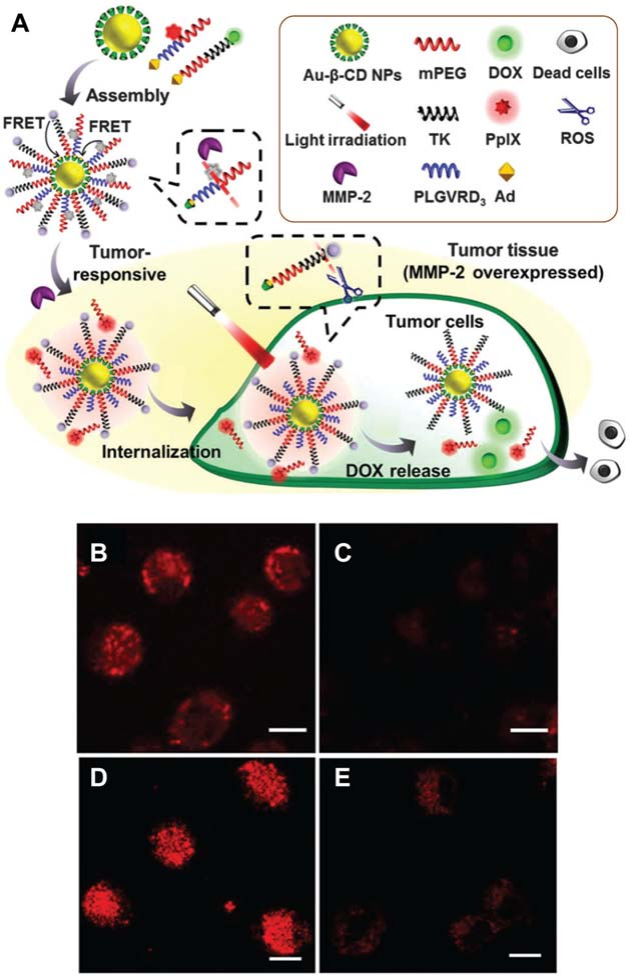
(A) Schematic illustration of multiple‐stimuli responsive NPs for light triggered drug release and PDT. (B–E) Confocal fluorescence images of cells incubated with Au/PpIX/DOX NPs showing fluorescence recovery for PpIX in SCC‐7 cells (B) and COS7 cells (C) and for DOX in SCC‐7 cells with (D) and without (E) light irradiation for 30 min. The scale bar represents 10 μm for all images. Adapted from Ref. 49 with permission. Copyright the Royal Society of Chemistry 2015

In addition to dual‐responsive system, DDS with multi‐responsiveness to light, ROS and enzymes were also reported based on inorganic NPs. In one example, gold NPs (AuNPs) were used as carrier and fluorescence quencher for FRET based tumor imaging and light manipulated on‐demand drug release.^49^ As shown in Figure 4A, PEG tethered with a PS (PpIX) is conjugated to β‐Cyclodextrin‐SS (b‐CD‐SS) modified AuNPs via an MMP‐2 responsive peptide linker (PLGVR). The NPs are further anchored with DOX via a ROS‐responsive thioketal linker. The fluorescence of both PpIX and DOX is quenched by AuNPs via FRET. The functionalized AuNPs can selectively target tumor tissue with overexpressed MMP‐2, and the cleavage of the peptide linker recovers the quenched fluorescence of PpIX for cell imaging. Upon cell internalization and subsequent light irradiation, thioketal linker breaks and releases DOX to allow for combined chemotherapy and PDT. The two‐step fluorescence recovery for PpIX and DOX was demonstrated in Figure 4B–E. The fluorescence of PpIX in SCC‐7 cancer cells (Figure 4B) is much stronger than that in COS7 fibroblast cells (Figure 4C) due to the targeting ability of the NPs to SCC‐7 with high MMP‐2 expression. Much stronger fluorescence from DOX was observed in SCC‐7 cells upon light irradiation for 30 min in comparison to that without light irradiation (Figure 4D,E), indicating effective light‐activated drug release. As expected, the Au/PpIX/DOX NPs cause much higher cytotoxicity to SCC‐7 cells than COS7 cells due to the combined effect of targeting, PDT and chemotherapy and the toxicity increases with increasing PS concentration.

Another example is based on a multifunctional ZnO cocktail for combinatorial therapy.^50^ Multiple elements were carried to achieve different tasks: hyaluronic acid was employed to target CD44 receptor and respond to tumor rich hyaluronidase; DOX was chosen as chemotherapeutic agent; cell penetrating peptide served to enhance cell uptake; ZnO could degrade at acidic environment to release toxic Zn^2+^ and ROS. In vivo study of the system showed that significant apoptosis was induced by the cocktail (71.2 ± 8.2%) as compared to free DOX (12.9 ± 5.2%).

### 3.3 ROS‐ and pH‐responsive DDSs

**Figure 5:**
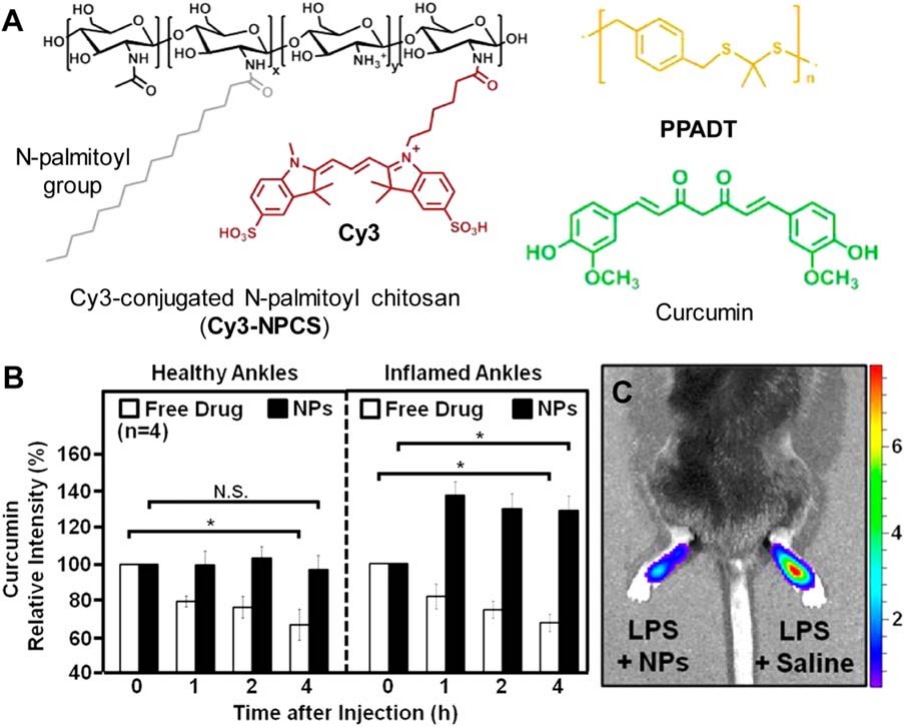
(A) Composition of the pH and ROS dual‐responsive DDS and chemical structures of the components: Cy3 conjugated NPCS, PPADT, and curcumin. (B) Relative fluorescence intensities of curcumin for healthy and inflamed ankles treated with free‐form curcumin and the curcumin‐loaded NPs. (C) IVIS image of mouse injected with L‐012 with LPS‐stimulated ankles after treatment with NPs and saline. Adapted from Ref. 51 with permission. Copyright 2014 American Chemical Society

Acidic pH is one of the key characteristics of pathological conditions including tumor, inflammation and organelles like endo/lysosome. In a typical ROS and pH dual‐responsive system, the pH responsive elements generally improve drug release by inducing a morphology change of the drug carrier upon protonation or deprotonation. Based on this strategy, a ROS and pH dual‐responsive system was reported for drug delivery to inflammatory areas with oxidative stress and reduced pH conditions.^51^ The DDS involves a Cy3‐labelled pH‐responsive N‐palmitoyl chitosan (NPCS) which forms NP with a polythioketal and therapeutic agent curcumin (Figure 5A). On one hand, the encapsulated hydrophobic polythioketal is degraded to hydrophilic fragments, which causes NP disintegration. On the other hand, the low pH causes protonation of amine group in NPCS and subsequent morphology change of NPCS that favors NP dissociation. The mechanism of anti‐inflammatory effect is attributed to both extracellular radical scavenging and intracellular inhibition which downregulates the pro‐inflammatory cascades. Cy3 and curcumin serve as a FRET pair for monitoring of curcumin release behaviors and oxidant inhibitory effect. It was found that 50% of encapsulated curcumin can be released in 4 hr in the presence of 1 mM H_2_O_2_ at pH 5.5 and the H_2_O_2_ level in LPS‐stimulated macrophage was reduced from 2.6‐fold to 1.1‐fold upon incubation with curcumin loaded NPs for 4 hr. To gain insight into biodistribution, the florescence of curcumin was collected from healthy and inflamed mouse ankles injected with either free drug or curcumin loaded NPs as a function of time after injection (Figure 5B). Results show that the curcumin‐NPs can be better retained than free drugs and the increasing intensity for curcumin‐NP in inflamed ankles indicates effective drug release triggered by oxidative stress and lowered pH in the inflamed condition. A luminescent probe L‐012 was intravenously administered to detect the ROS level in vivo. The much lower chemiluminescence at the inflamed ankle with curcumin‐NP treatment than that with saline treatment indicates much reduced ROS level and good inhibitory effect for the former (Figure 5C).

Oligoproline was also found to be cleaved by ROS species (Scheme 1). Early in 2011, polymeric scaffolds cross‐linked with proline oligomers were tested by Sung's group for oxidation responsiveness as a potential drug delivery carrier.^52^ The same group later reported a polyproline‐containing pH liable block terpolymer for gene delivery.^53^ The terpolymer contains an equimolar ratio of positively charged block and negatively charged block at physiological pH. The polymer was further complexed with plasmid DNA for pathological vascular targeted gene delivery. At reduced pH in endosomes, protonation of the negatively charged block caused destabilization of the polymer core and membrane disruption, leading to endomal escape of the nanocarrier. Subsequent exposure to excessive ROS in vascular smooth muscle cells further degrades the polymer to enable pDNA release.

When subject to pH stimuli, the DDS can be directly cleaved to release the cargo. Early in 2010, polyoxylate materials have been explored as vehicle for controlled drug release.^54^ The polymer can be degrade hydrolytically by H_2_O_2_ to yield oxalic acid and 1,4‐cyclohexanedimethanol. The polymer NPs were found to degrade in RAW 264.7 macrophage cells and HEK 293 (human embryonic kidney) cells in a time‐ and dose‐dependent manner. Later in 2013, a polymeric prodrug of vanillin, potent antioxidant and anti‐inflammatory agent, was prepared with ROS‐responsive peroxalate ester bond and vanillin via acid‐responsive acetal linkages in its backbone.^55^ Under inflammation conditions, the excessive ROS will react with peroxalate ester bond while acidic pH will cleave the acetal linkages to release vanillin. As a result, the therapeutic effect is attributed to both the ROS scavenging ability of the NP and the anti‐inflammatory effect of vanillin. Intravenous administration of the prodrug lowered the expression of pro‐inflammatory cytokines in activated macrophages and significantly declined the acetaminophen‐induced acute hepatic injury. Another work also reported an ortho ester‐ and boronic ester‐containing block copolymers which degrades via a combination effect of ortho ester hydrolysis and boronic ester oxidation.^56^ Both processes were accelerated by increasing amount of H_2_O_2_ and the acceleration is highly pH dependent.

Less commonly, change of pH may lead to charge reversion of DDS to facilitate cell internalization as positively charged NPs are preferably taken up by cells. Recently, Dong's group also demonstrated a pH and ROS dual‐responsive DDS for intracellular drug delivery.^57^ The carrier consists of a block copolymer of PEG and polycaprolactone connected via a thioether linker. An acid‐labile b‐carboxylic amide segments with charge reversal properties were tagged along the polycaprolactone side chain. The amphiphilic block copolymer self‐assembled to form NPs and DOX was encapsulated into the NPs through hydrophobic interaction. While the charge reversion of the drug NP from negative to positive favored effective internalization by acidic tumor, the ROS‐responsive linker led to accelerated drug release in response to high ROS (H_2_O_2_) level in the tumor.

As discussed so far, the major delivery mechanism is based on chemical bond cleavage or solubility switch that degrades drug carrier. A recent work reports a novel DDS that releases drug upon shell disruption by gas bubbles generated in response to both ROS exposure and in situ created acidic miliew.^58^ The DDS consists of a poly lactic‐*co*‐glycolic acid (PLGA) microsphere that carries Dexamethasone sodium phosphate (DEX‐P) as anti‐inflammatory drug, ethanol and FeCl_2_ as acid precursor, and sodium bicarbonate (SBC) as gas generating agent. When reaching inflamed steoarthritis, the overproduced H_2_O_2_ penetrated the microsphere to oxidize ethanol in the presence of Fe^2 +^ *via* Fenton reaction, creating an acidic environment. Subsequently, SBC decomposed under acidic conditions and generated CO_2_ gas bubbles which caused burst microsphere shell and release of DEX‐P. The DDS was demonstrated to have efficient anti‐inflammatory effect that protects against joint destruction in mouse model.

### 3.4 ROS‐ and thermal‐responsive DDSs

**Figure 6:**
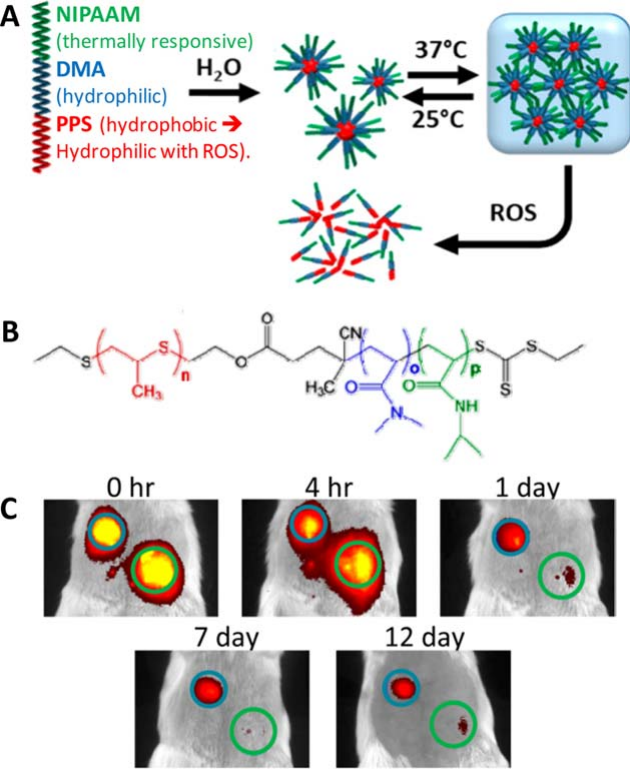
(A) Schematic illustration of gelation of triblock copolymer at 37 °C and ROS‐induced disassembly. (B) Chemical structure of polymer PPS‐DMA‐NIPAAM. (C) IVIS images of mouse subcutaneously injected with 50 μL of dye‐loaded triblock polymer solution (blue circle, top left) and dye‐loaded diblock copolymer solution (green circle, bottom right). Adapted from Ref. 59 with permission. Copyright 2014 American Chemical Society

Temperature is another common stimuli that has been widely investigated in oncology. In view of the slightly higher temperature of tumor microenvironment than that of normal tissues, thermal‐responsive materials are designed to collapse in response to elevated temperature in tumor or upon externally induced local hyperthermia to release its payload. The temperature difference between ambient and physiological conditions may also require thermal‐responsive materials for drug administration or loading purposes. A thermoresponsive hydrogel based on PPS containing triblock polymer was reported for temperature modulated ROS‐triggered drug release.^59^ As shown in Figure 6A,B, the ABC triblock polymer consists of three parts: the thermal‐responsive *N*‐isopropylacrylamide (NIPAAM), hydrophilic *N,N*‐dimethylacrylamide (DMA) and hydrophobic ROS‐responsive propylenesulfide (PPS), which self‐assembled into 66 ± 32 nm micelles at ambient temperature (25 °C). The polymer micelles underwent a sharp transition to cross linked gel structure when reaching physiological temperature of 37 °C that is above the lower critical solution temperature (LCST) of PNIPAAM. The hydrogel is expected to degrade upon exposure to ROS due to solubility switch of the PPS component that causes micelle disassembly. The hydrogel loaded with model drug Nile red showed increased drug release in the presence of H_2_O_2_ by monitoring Nile red fluorescence change and exhibited H_2_O_2_‐dependent drug release kinetics. Importantly, the polymer hydrogel without drug was found to cause minimal cytotoxicity and showed cytoprotective effect against H_2_O_2_ for incubated NIH 3T3 mouse fibroblasts cells, which is attributed to the ROS scavenging capability of PPS.^60^ Finally, the Nile red loaded hydrogel was injected subcutaneously into male BALB/c mice to monitor local retention of the drug released. As shown in Figure 6C, the drug loaded triblock hydrogel provides a sustained local release over two weeks, whereas the control with diblock (withought NIPAAM) polymer shows rapid drug diffusion and poor retention.

Another example of ROS and thermal dual‐responsive DDS was reported by Chen's group.^61^ The triblock polymer consists of alternating polyethylene glycol (PEG) as the shell and a thermal and oxidation dual‐responsive thioether containing polymer as the core. The hydrophobic drugs such as Nile red are encapsulated into the collapsed carrier at elevated temperature and released upon ROS exposure.

### 3.5 Dual redox‐responsive DDSs

The intracellular environment is known to have high reduction level due to presence of reducing agents such as glutathione (GSH) (0.5–10 mM). The GSH level in tumor cells is several fold higher than the normal ones, making it a useful stimuli for targeting tumor cells and triggering drug delivery.^62^ Two types of materials have been reported for redox‐responsive DDS.

Diselenides generally exhibit dual redox‐responsive properties. The Se‐Se bond can either be oxidized to seleninic acid by ROS or be reduced to selenol by reducing agents. A triblock copolymer micelle system (PEG‐PUSeSe‐PEG) with a polyurethane (PU) block containing diselenide and two blocks of PEG were demonstrated for dual response to both H_2_O_2_ and GSH.^63^ The amphiphilic block copolymer self‐assembled in aqueous solution to form NPs of ∼76 nm size. Either oxidation or reduction will lead to polymer chain dissociation and NP disassembly. The cargo release behavior was studied using Rhodamine B (RB) loaded NPs. It took ∼1.1 hr and ∼2 hr to release 80% RB in the presence of 0.1% H_2_O_2_ (equivalent to 30 mM) or 0.1 mg/mL GSH, respectively. Later in 2014, the group further developed a general strategy using a phospholipid and diselenide‐containing block copolymer that form coassemblies by electrostatic interaction for dual redox response.^64^

**Figure 7:**
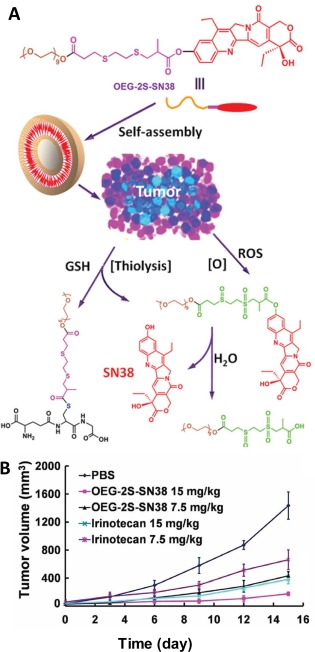
(A) Chemical structure of the prodrug and schematic illustration of self‐assembly and drug release via both thiolysis and ROS oxidation. (B) Plot of tumor volume of mice treated with OEG‐2S‐SN38 and irinotecan at different dosages versus time. Adapted from Ref. 65 with permission. Copyright 2013 Wiley‐VCH Verlag GmbH & Co. KGaA, Weinheim

A thioether containing DDS responsive to both glutathione (GSH) and ROS was also reported for tumor therapy.^65^ To construct such a tumor heterogeneity‐responsive system, both a GSH‐responsive phenol ester linker and ROS‐responsive thioether linker were used. As shown in Figure 7A, the prodrug consists of a camptothecin‐based topoisomerase I inhibitor 7‐ethyl‐10‐hydroxyl‐camptothecin (SN38) conjugated with the PEG via the phenol ester and thioether linkers, which then self‐assembles to form nanocapsules (OEG‐2S‐SN38) with ∼100 nm diameter. When internalized by tumors that are rich in both GSH and ROS, the nanocapsules can either undergo thiolysis or ROS‐triggered hydrolysis of phenol ester to release SN38. Results revealed that 80% SN38 could be released within 15 min in the presence of 10 mM, which is much faster than the diselenides mentioned earlier. Two hour was needed to release 80% SN38 in the presence of 10 mM GSH at pH 7.4. The nanocapsules were tested with Bcap37 (BC) cells, showing enhanced in vitro cytotoxicity, which was proved to result from triggered SN38 release. In vivo antitumor activities were also studied using Bcap37 breast tumor xenograft model for OEG‐2S‐SN38a and a clinically used SN38 prodrug irinotecan. Figure 7B shows the tumor volume of mice treated with PBS and two prodrugs, indicating a much higher inhibition rate achieved by OEG‐2S‐SN38a prodrug, which is attributed to the combination of enhanced permeation and retention (EPR) effect and therapeutic effect of released SN38 drug.

## 4 CONCLUSIONS

Among the many internal stimuli, ROS represents a unique signature for many pathological conditions, making it an attractive trigger for drug release. Although ROS‐responsive DDSs have only emerged in recent years, they have already demonstrated huge potential in biomedical applications. Various examples of ROS‐responsive DDSs have been discussed in this review, including delivery of small molecule drugs, nucleic acids and proteins for applications in cancer therapy, anti‐inflammation and vaccination.

Depending on the nature of payloads, suitable drug carriers can be selected to load drugs via hydrophobic interaction, electrostatic interaction and covalent bonding, which allow drugs to be released via different mechanisms. DDSs with polymers containing thioether and monoselenium or monotellurium generally release encapsulated drug based on a phase change induced carrier disassembly and they have increased ROS sensitivity with increasing electronegativity. Polymers containing diselenides and oxylate are usually degraded into small pieces to release the cargo. Other materials based on boronic ester, thioketal and aminoacrylate are frequently used as ROS‐cleavable linker which directly liberate the drugs linked. While no direct relationship has been established between drug release efficiency and the type of ROS‐responsive material, it is generally believed that the carrier material with high sensitivity to ROS and reaction with easy access of ROS that leads to direct drug release (such as DDS with ROS‐linker conjugated drug) will result in higher release efficiency.

Multiple‐stimuli responsive DDSs that are also sensitive to other internal stimuli such as pH, temperature, enzymatic activity and reducing agents hold the promise to offer better targeting ability to diseased sites. In addition to target sites that overproduce ROS, the ROS‐responsive DDS may also be applied to other sites of interest by introduction of a PS that can generate ROS in situ upon light irradiation. The PS not only allows for light triggered drug release, but also kills cells or bacteria though PDT, enabling combinatorial therapy with improved therapeutic efficiency. Multifunctional DDS that combines therapy with imaging capability is beneficial for revealing the spatial and temporal location of the drug, facilitating the pharmacokinetic study and early diagnosis of disease. It is believed that the applications of the DDS can be greatly expanded by recruitment of multifunctional elements such as targeting ligand, imaging contrast agents, for theranostic platform, multi‐modal therapy and multiple‐stimuli sensitive drug delivery applications. It is hoped that this review will inspire the development more advanced DDS and clinically relevant applications toward translational medicine.

## CONFLICT OF INTERESTS

The co‐authors of this article do not have a conflict of interest to declare.
